# Anion Inhibition Studies of the β-Class Carbonic Anhydrase CAS3 from the Filamentous Ascomycete *Sordaria macrospora*

**DOI:** 10.3390/metabo10030093

**Published:** 2020-03-05

**Authors:** Daniela Vullo, Ronny Lehneck, William A. Donald, Stefanie Pöggeler, Claudiu T. Supuran

**Affiliations:** 1 Dipartimento di Chimica Ugo Schiff, Università degli Studi di Firenze, 50019 Sesto Fiorentino (Florence), Italy; daniela.vullo@unifi.it; 2 Institute of Microbiology and Genetics, Department of Genetics of Eukaryotic Microorganisms, Georg-August-University Göttingen, 37077 Gottingen, Germany; ronnylehneck@hotmail.de; 3 University of New South Wales, School of Chemistry, Sydney, NSW 2052, Australia; w.donald@unsw.edu.au; 4 Neurofarba Dept., Section of Pharmaceutical and Nutriceutical Sciences, Università degli Studi di Firenze, 50019 Sesto Fiorentino (Florence), Italy

**Keywords:** carbonic anhydrase, fungus, anions, small molecules, antifungals, *Sordaria macrospora*

## Abstract

CAS3 is a newly cloned cytosolic β-class carbonic anhydrase (CA, EC 4.2.1.1) from the filamentous ascomycete *Sordaria macrospora*. This enzyme has a high catalytic activity for the physiological CO_2_ hydration reaction and herein, we report the inhibition profile of CAS3 with anions and small molecules. The most effective CAS3 anions/small molecule inhibitors were diethyl-dithiocarbamate, sulfamide, sulfamate, phenyl boronic and phenyl arsonic acids, with K_I_s in the range of 0.89 mM–97 µM. Anions such as iodide, the pseudohalides, bicarbonate, carbonate, nitrate, nitrite, hydrogensulfide, stannate, selenate, tellurate, tetraborate, perrhenate, perruthenate, selenocyanide and trithiocarbonate were low millimolar CAS3 inhibitors. The light halides, sulfate, hydrogensulfite, peroxydisulfate, diphosphate, divanadate, perchlorate, tetrafluoroborate, fluorosulfonate and iminodisulfonate did not significantly inhibit this enzyme. These data may be useful for developing antifungals based on CA inhibition, considering the fact that many of the inhibitors reported here may be used as lead molecules and, by incorporating the appropriate organic scaffolds, potent nanomolar inhibitors could be developed.

## 1. Introduction

Anions constitute an important class of inhibitors for many metalloenzymes due to their capacity to bind to the metal ion within their enzyme active site, which thereafter interferes with the catalytic process [1,2,3,4,5]. Anion inhibitors, although usually not highly effective, are relevant both for understanding, in detail, the inhibition mechanisms of metalloenzymes and for drug design purposes. This is because such simple chemical entities are easily modifiable for developing more elaborate scaffolds which leads to highly effective inhibitors. An example of this type of small molecules inhibitor is trithiocarbonate (CS_3_^2-^), a weak, millimolar anion inhibitor [6] of the zinc enzyme carbonic anhydrase (CA, EC 4.2.1.1) [7,8], which led to the discovery of three effective, nanomolar classes of CA inhibitors (CAIs), the dithiocarbamates [9], the monothiocarbamates [10] and the xanthates [11]. Trithiocarbonate has a K_I_s in the range of 8.7–9.9 µM for human (h) isoforms hCA I-III, 36.15 mM for hCA VII and 0.43 mM for hCA XIII [6], whereas the three classes of organic inhibitors achieved low nanomolar inhibition for pharmaceutically relevant CA isoforms, such as hCA I, II, IV, IX and XII [9,10,11,12,13]. Thus, many anion inhibitors have been investigated for their interaction with CAs from various organisms, such as mammals (humans) [4,7,8,14], bacteria [15,16,17,18], protozoans [19,20,21], corals [22] fungi [23,24,25,26,27] and Archaea [28].

Fungi encode for CAs belonging to the α- and β-CA genetic families (of the eight CA classes presently known [29]) and these enzymes play crucial roles in the growth, development, virulence and survival of these organisms [30,31]. Modulation of their activity with inhibitors and/or activators has been proposed as a new approach for designing antifungals [32,33,34,35].

The filamentous ascomycete *Sordaria macrospora* is a coprophilous fungus that naturally lives on herbivore dung and was shown to encode for at least three β- and one α-class CAs [24,30,36,37,38,39,40]. Two of the β-CAs, CAS1 and CAS2, and their X-ray crystal structures [24], were investigated in detail by some of us. Sulfonamide [34] inhibition profiles have also been reported. Unlike other fungal β-CAs, CAS1 and CAS2 were shown to be tetrameric enzymes (dimers of dimers), possessing a rather moderate CO_2_ hydrase activity. They are moderately inhibited by most anions and sulfonamides [24,34]. As *S. macrospora* is a model organism for studying the fruiting-body development in fungi [36], and as it has been shown that some of the CAs encoded in its genome are involved in biosynthetic pathways, as in other patogenic fungi (e.g., *Candida* spp. [25,27] and *Cryptococcus neoformans* [41]), it appeared of interest to study in more detail the CAs encoded in its genome.

Here, we report that CAS3, the third β-CA found in the *S. macrospora* genome, is a highly active catalyst for the physiologic CO_2_ hydration reactions and we present the first anion inhibition study of this enzyme, comparing it to those of CAS1 and CAS2 investigated earlier [24]. Our findings may be relevant for developing alternative antifungals to those that are clinically used, for which extensive resistance has been documented [42].

## 2. Results and Discussion

**Figure 1:**
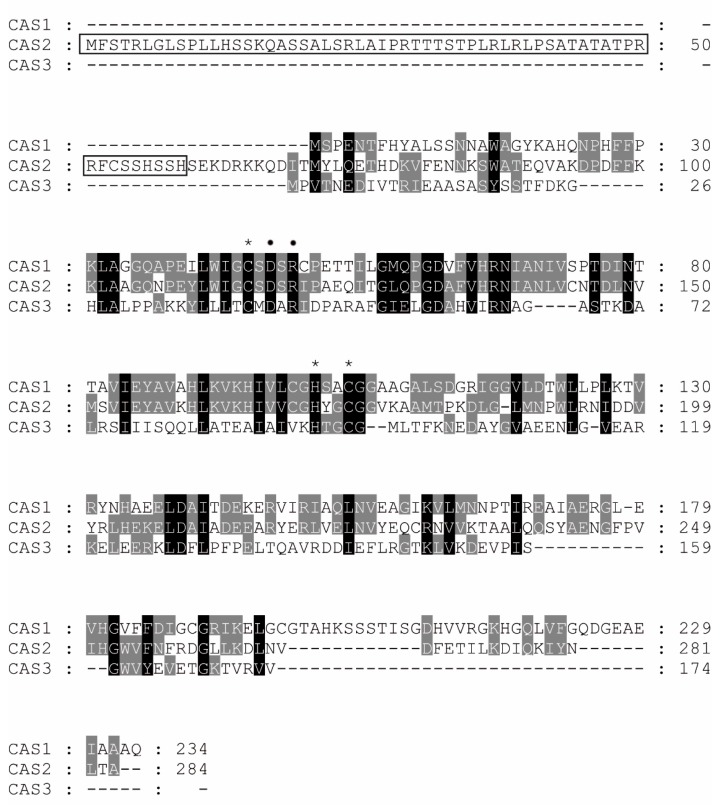
Alignment of three β-class CAs from *Sordaria macrospora*. Sequences were aligned to using ClustalX [43]. Residues, which are conserved in all three sequences, are indicated in white and highlighted in black, and identical amino acids in the two sequences are highlighted in grey. Conserved amino acids that are predicted to be important for zinc-coordination (Cys41, His93 and Cys96 in CAS3) are marked by an asterisk, and residues of the catalytic dyad (Asp43 and Arg45 in CAS3) are marked by a dot. Amino acid positions are given as numbers at the right side of the alignment. The N-terminal mitochondrial target sequence (aa 1–59) of CAS2 is boxed.

The *cas3* gene encodes for a 174 amino acid residue protein, CAS3, which is the shortest of the three β-CAs found in *S. macrospora* [37,38]. As seen in Figure 1, CAS1 is a 234 amino acid protein and CAS2 has 284 residues, whereas CAS3 is much shorter, as described above.

**Figure 2:**
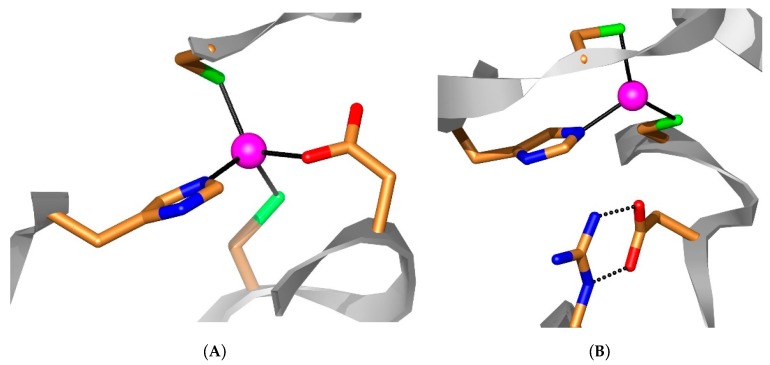
Active site architecture of a generic β-CA: (**A**) a closed-active site enzyme [45,46,47] with the zinc ion (**magenta sphere**) coordinated by a His residue (**in blue**), two Cys residues (**in green**) and an Asp residue of the catalytic dyad (**in red**). (**B**) Open active site. The His and Cys residues remain coordinated to the Zn(II) ion whereas the Asp makes a pH dependent salt bridge interaction with the Arg of the dyad. Thus, the fourth coordination position of Zn(II) will be occupied by a water molecule (**not shown**). In the case of CAS3, Cys41, His93 and Cys96, the residues are shown in panels A and B, and the Asp43 in panel A is coordinated to the Zn(II) ion or forms a salt bridge with Arg45 in panel B.

However, an alignment of the three proteins reveals that as all β-Cas and CAS3 have the features characteristic of an enzymatically active CA, i.e., (i) three zinc ion residues (Cys41, His93 and Cys96, CAS3 numbering system, see Figure 1), with the fourth coordination site presumably being occupied by a water molecule/hydroxide ion; and (ii) the catalytic dyads Asp43 and Arg45 (CAS3 numbering system), conserved in all known β-CAs and involved in activating the zinc-bound water molecule for catalysis [27,41]. In contrast, for the closed active site β-CAs [15,44,45], the Arg is also involved in the “opening” of the active site, as documented for the *Mycobacterium tuberculosis* enzymes [46,47] and shown in Figure 2. On the other hand, as observed in Figure 1, there are many conserved amino acid residues among the three enzymes, except for the amino-terminal and carboxy-terminal parts, in which both the length of the polypeptide and the composition are rather different.

Thus, we investigated the CO_2_ hydrase activity of CAS3 and compared it to that of other α- and β-class enzymes, as shown in Table 1.

CAS3 has been shown earlier [37,38] to belong to the Cab-type β-CAs. As Cab has an open active site [28,48], we assume that this is also the case for CAS3. However, all kinetic and inhibition measurements were performed at a pH value of 8.3, in which the active site should be opened even for the closed active site of β-CAs [45,46,47]. The activity of CAS3 was compared to that of other β-CAs from fungi/yeasts/archaea (Can2 from *Cryptococcus neoformans* [41], CalCA from *Candida albicans* [27], SceCA from *Saccharomyces cerevisiae* [26]), Cab from as *Methanobacterium thermoautotrophicum* [28], CAS1 and CAS2 from *S. macrospora* [24]), as well as with the widespread α-class human isoforms hCA I and II [7]. As seen from the data of Table 1, CAS3 shows an order of magnitude higher catalytic activity compared to CAS1, CAS2 and Cab, with the following kinetic parameters: k_cat_ of (7.9 ± 0.2) × 10^5^ s^−1^, and k_cat_/K_m_ of (9.5 ± 0.12) × 10^7^ M^−1^ × s^−1^. The activity of CAS3 is thus similar to that of CalCA and SceCA, being almost two times higher than that of the “slow” human isoform hCA I, a highly abundant enzyme in red blood cells [27]. The most effective among these enzymes is hCA II, a “perfectly” evolved catalyst [49].

With this active enzyme in hand, we performed an anion inhibition study of CAS3, with a rather large number of simple and complex inorganic anions, as well as several small molecules known to inhibit CAs, such as sulfamide, sulfamic acid, phenyl boronic and phenyl arsenic acids [4,24,25,26,27,28,29,30] (Table 2).

As shown in Table 2, and as expected, most of the investigated anions/small molecules act as weak–medium potency CAS3 inhibitors. The following results will be highlighted as they seem to provide interesting information on the features of this enzyme:

(i) The following anions did not significantly inhibit CAS3 up to a concentration of 100 mM in the assay system: the halides (except iodide); sulfate, hydrogensulfite and peroxydisulfate; diphosphate and divanadate; perchlorate, tetrafluoroborate, fluorosulfonate and iminodisulfonate (Table 2). Some of the anions, such as perchlorate and tetrafluoroborate, are known to possess a low affinity for binding metal ions, both in solution and within the metalloenzyme active sites [4]. However, the halides generally inhibit CAs, as seen in Table 2 in which millimolar inhibition was reported for CAS1 and hCA II (except fluoride).
(ii) Most of the investigated anions were low millimolar, weak CAS3 inhibitors, with inhibition constants in the range of 3.2–9.9 mM. They include iodide, the pseudohalides, bicarbonate, carbonate, nitrate, nitrite, hydrogensulfide, stannate, selenate, tellurate, tetraborate, perrhenate, perruthenate, selenocyanide and trithiocarbonate.
(iii) Submillimolar inhibition was observed with diethylditiocarbamate (K_I_ of 0.89 mM), as well as sulfamide, sulfanate, phenyl boronic and phenyl arsonic acids (K_I_s of 91–97 µM). As expected, the weak inhibitor trithiocarbonate (K_I_ of 8.6 mM) led to an order of magnitude more effective inhibitor when the diethylamino fragment was introduced, with *N*,*N*-diethyl-ditiocarbamate showing a K_I_ of 0.89 mM (Table 2).
(iv) The inhibition profile of CAS3 is rather different from that of the related isoforms, CAS1 and CAS2, and also from hCA II. However, no CAS3-selective inhibitors have been detected so far among these simple anions and small molecules. On the other hand, the identification of submillimolar or high micromolar inhibitors (diethyl-ditiocarbamate, sulfamide, sulfamate, phenyl boronic and phenyl arsonic acids) shows that it is probably possible to design compounds with an enhanced activity. In fact, acetazolamide (5-acetamido-1,3,4-thiadiazole-2-sulfonamide), which incorporates the sulfonamide fragment present in sulfamide and sulfamic acid, is a nanomolar CAS3 inhibitor (Table 1).

## 3. Materials and Methods

### CA Activity and Inhibition Measurements

An Applied Photophysics stopped-flow instrument was used for assaying the CA catalyzed CO_2_ hydration activity [44]. Phenol red or bromothymol blue (at a concentration of 0.2 mM) were used as indicators following the initial rates of the CA-catalyzed CO_2_ hydration reaction for a period of 10–100 s. The indicators worked at the absorbance maximum of 557 nm, with 10–20 mM TRIS (pH 8.3) as buffer, and 10 mM NaClO_4_ for maintaining the ionic strength. The CO_2_ concentrations ranged from 1.7 mM to 17 mM for the determination of the kinetic parameters and inhibition constants. For each inhibitor, at least six traces of the initial 5%–10% of the reaction were used for determining the initial velocity. The uncatalyzed rates were determined in the same manner and subtracted from the total observed rates. Stock solutions of inhibitors (10 mM) were prepared in distilled-deionized water and dilutions up to 0.01 µM were done thereafter with distilled-deionized water. Inhibitor and enzyme solutions were preincubated together for 15 min at room temperature prior to the assay in order to allow for the formation of the E–I complex. The inhibition constants were obtained by the non-linear least-squares method using PRISM 3 and the Cheng–Prusoff equation, whereas the kinetic parameters for the uninhibited enzymes from Lineweaver–Burk plots, as reported earlier [24,25,26,27,28,29], represent the mean from at least three different determinations. All inhibitors (sodium salts of the anions from Table 2, as well as the other small molecules) were commercially available, highest purity compounds from Sigma-Aldrich (Milan, Italy) and were used as received. CAS3 has been obtained in recombinant form [50], as reported earlier for the related isoforms CAS1 and CAS2 [24,34]. In particular, production of the CAS3 protein was performed in E. coli strains, Rosetta (DE3) (Invitrogen, Germany). An overnight pre-culture was used to inoculate 4 × 0.5 L of Luria broth (LB) medium supplemented with 100 mg/L ampicillin and 0.5 mM ZnSO_4_. The cultures were grown to an OD600 of 0.1. Heterologous gene expression was then induced by the addition of 1 mM IPTG during the exponential phase of growth and lasted for 3–4 h at 30 °C. Subsequently, the cells were harvested (4000 g, 30 min, 4 °C) and flash frozen with liquid nitrogen and stored at −20 °C. For purification of CAS3, 10 g of *E. coli* cells were re-suspended in 30 mL lysis buffer (20 mM imidazole, 50 mM NaH_2_PO_4_ pH 8, 300 mM NaCl, 0.02 mM MgCl_2_, 1 “Protease Inhibitor Cocktail” Tablet (Roche, Germany)) and subsequently incubated for 30 min at 4 °C with a spatula tip of lysozyme (Serva, Germany, 28,262.03) and DNase I. After incubation, the cells were disrupted using a microfluidizer 110S (Microfluids, Germany). After centrifugation (50,000 × *g*, 30 min, 4 °C), the clarified lysate was applied on a Ni-NTA agarose (Qiagen, Germany, 1,018,244) column equilibrated with lysis buffer. Unbound proteins were removed by washing gradually with 3 column volumes (CV) of lysis buffer containing 40 mM, 60mM, 80 mM and 100 mM imidazole, respectively. The bound CAS3 was eluted with lysis buffer containing 250 mM imidazole. Elution fractions were analyzed by SDS-PAGE, pooled, concentrated in a “Spin-X^®^ UF 20” (Corning, Germany) and stored at 4 °C. After purification, 5–7.5 mg of CAS3 could be obtained per L of culture [50].

## 4. Conclusions

Fungal CAs are of great interest for both biotechnological and pharmaceutical applications [30], since some of these enzymes are stable, relatively easy to produce and may be used as model enzymes for testing inhibitors/activators. *Sordaria macrospora,* an organism used as a genetic model to study fruiting body development of filamentous fungi, encodes for at least four different CAs, two of which, CAS1 and CAS2, have been thoroughly investigated earlier. Here, we proved that CAS3, another representative enzyme from this organism, belonging to the β-CA class, may be of interest for better understanding the roles these proteins play in various physiologic processes of fungi. Unlike CAS1 and CAS2 which showed rather low catalytic activity for the hydration of CO_2_ to bicarbonate and protons, CAS3 is a highly effective catalyst, showing kinetic parameters comparable to those of other fungal/mammalian enzymes, i.e., k_cat_ of (7.9 ± 0.2) × 10^5^ s^−1^ and k_cat_/K_m_ of (9.5 ± 0.12) × 10^7^ M^−1^ × s^−1^. A detailed inhibition study of CAS3 with anions and other small molecules known to bind to metalloenzymes is reported here. The most effective anions/small molecule inhibitors were diethyl-ditiocarbamate, sulfamide, sulfamate, phenyl boronic and phenyl arsonic acids, with K_I_s in the range of 0.89 mM–97 µM. Anions such as iodide, the pseudohalides, bicarbonate, carbonate, nitrate, nitrite, hydrogensulfide, stannate, selenate, tellurate, tetraborate, perrhenate, perruthenate, selenocyanide and trithiocarbonate were low millimolar inhibitors. The light halides, sulfate, hydrogensulfite, peroxydisulfate, diphosphate, divanadate, perchlorate, tetrafluoroborate, fluorosulfonate and iminodisulfonate did not significantly inhibit this enzyme. These data may be useful for developing antifungals based on CA inhibition, considering the fact that many of the relatively effective inhibitors reported here may be used as lead molecules and, by adding the appropriate scaffolds, they could generate potent, nanomolar inhibitors.

## Figures and Tables

**Table 1:** Kinetic parameters for the CO_2_ hydration reaction [44] catalyzed by the three CAS enzymes. CAS1-CAS3, the human cytosolic isozymes hCA I and II (α-class CAs) at 20 °C and pH 7.5 in 10 mM HEPES buffer (for the α-CAs), as well as Can2 (from *C. neoformans*), CalCA (from *C. albicans*), SceCA (from *S. cerevisiae*) and Cab (from the archaeon *Methanobacterium thermoautotrophicum*) measured at 20 °C, pH 8.3 in 20 mM TRIS buffer and 20 mM NaClO_4_. (for all β-CAs). Inhibition data with the clinically used sulfonamide acetazolamide (5-acetamido-1,3,4-thiadiazole-2-sulfonamide) are also provided.

Isozyme	Activity Level	k_cat (s_^−1^_)_	k_cat_/Km (M^−1^ × s^−1^)	K_I_ (Acetazolamide) (nM)
hCA I ^a^	moderate	2.0 × 10^5^	5.0 × 10^7^	250
hCA II ^a^	very high	1.4 × 10^6^	1.5 × 10^8^	12
Can2 ^b^	moderate	3.9 × 10^5^	4.3 × 10^7^	10.5
CalCA^c^	high	8.0 × 10^5^	9.7 × 10^7^	132
SceCA ^d^	high	9.4 × 10^5^	9.8 × 10^7^	82
Cab ^e^	low	3.1 × 10^4^	1.82 × 10^6^	12,100
CAS1 ^f^	low	1.2 × 10^4^	1.30 × 10^6^	445
CAS2 ^f^	low	1.3 × 10^4^	1.21 × 10^6^	816
CAS3 ^g^	high	(7.9 ± 0.2) × 10^5^	(9.5 ± 0.12) × 10^7^	94 ± 3

^a^ From refs. [7,8]; ^b^ From ref. [41]; ^c^ From ref. [27]; ^d^ From ref. [26]; ^e^ From ref. [28]; ^f^ From ref. [24]; ^g^ This work.

**Table 2:** Inhibition constants of anionic inhibitors against α-CA isozymes of human origin (hCA II), and the three β-CAs from *Sordaria macrospora,* CAS1, CAS2 and CAS3, at 20 °C by a stopped flow CO_2_ hydrase assay [44].

Inhibitor ^§^	hCA II ^a^	K_I_ [mM] # CAS1 ^b^	CAS2 ^b^	CAS3 ^c^
F^-^	>300	>100	>100	>100
Cl^-^	200	9.2	>100	>100
Br^-^	63	9.3	>100	>100
I^-^	26	8.6	7.7	9.9
CNO^-^	0.03	0.9	0.82	3.2
SCN^-^	1.6	5.4	5.6	7.3
CN^-^	0.02	0.94	0.75	8.7
N_3_^-^	1.51	>100	6.1	7.2
HCO_3_^-^	85	6.5	5.5	3.4
CO_3_^2-^	73	>100	8.8	8
NO_3_^-^	35	>100	>100	8.5
NO_2_^-^	63	>100	>100	8.3
HS^-^	0.04	0.89	8.5	8.3
HSO_3_^-^	89	3.3	7.3	>100
SO_4_^2-^	>200	>100	4.8	>100
SnO_3_^2-^	0.83	4.3	0.92	7.9
SeO_4_^2-^	112	2.4	9.2	3.4
TeO_4_^2-^	0.92	2.5	6.3	8.1
P_2_O_7_^4-^	48.5	3.1	0.96	>100
V_2_O_7_^4-^	0.57	>100	1.4	>100
B_4_O_7_^2-^	0.95	6.7	6.9	5.9
ReO_4_^-^	0.75	8.2	>100	8.8
RuO_4_^-^	0.69	3.9	>100	9.2
S_2_O_8_^2-^	0.084	5	>100	>100
SeCN^-^	0.086	2.9	9.3	7.1
CS_3_^2-^	0.0088	0.79	>100	8.6
Et_2_NCS_2_^-^	3.1	0.38	0.93	0.89
ClO_4_^-^	>200	> 100	> 100	>100
BF_4_^-^	>200	> 100	> 100	>100
FSO_3_^-^	0.46	0.93	8.4	>100
NH(SO_3_)_2_^2-^	0.76	0.88	9.2	>100
H_2_NSO_2_NH_2_	1.13	0.084	0.048	0.094
H_2_NSO_3_H	0.39	0.069	0.072	0.095
Ph-B(OH)_2_	23.1	0.009	0.056	0.097
Ph-AsO_3_H_2_	49.2	0.035	0.054	0.091

^§^ As sodium salts, except sulfamide and phenylboronic acid; # Errors were in the range of 3%–5% of the reported values from three different assays (data not shown).^a^ From refs. [7,8]; ^b^ From ref. [24]; ^c^ This work.
